# Prevalence of large vessel vasculitis in ANCA-associated vasculitis: a retrospective cohort study

**DOI:** 10.1007/s00296-021-04993-2

**Published:** 2021-09-24

**Authors:** Yann Coattrenec, Yannick D. Muller, David Spoerl, Johannes A. Lobrinus, Jörg D. Seebach

**Affiliations:** 1 Division of Immunology and Allergology, Department of Medicine, University Hospital and Medical Faculty, Rue Gabrielle Perret-Gentil 4; Geneva 14, 1211 Geneva, Switzerland; 2 Division of Immunology and Allergy, Centre Hospitalier Universitaire Vaudois, Rue du Bugnon 46, CH-1011 Lausanne, Switzerland; 3 Division of Clinical Pathology, Department Diagnostic, University Hospital and Medical Faculty, Geneva, Switzerland

**Keywords:** ANCA-associated vasculitis, Antineutrophil cytoplasmic antibodies, Large vessel vasculitis, Temporal arteritis, Aortitis, Granulomatosis with polyangiitis, Eosinophilic granulomatosis with polyangiitis, Microscopic polyangiitis

## Abstract

ANCA-associated vasculitis (AAV) in general involves small blood vessels and includes granulomatosis with polyangiitis (GPA), eosinophilic granulomatosis with polyangiitis (EGPA), and microscopic polyangiitis (MPA). Although reported in a few studies, the prevalence of large vessel vasculitis (LVV) in patients with AAV remains to be further explored. The goal of the present study was to assess the prevalence of LVV in a cohort of patients with AAV and to characterize this population. We conducted a ten-year retrospective study of a single-center cohort of AAV, including 101 patients with GPA (*n* = 58), EGPA (*n* = 28), MPA (*n* = 15), and compared the groups with or without associated LVV. LVV was diagnosed in five patients, two with aortitis and three with temporal arteritis, corresponding to a total prevalence of 5.0% [95% CI 1.6–11.2%]. This value was significantly higher than the estimated prevalence of LVV in the normal Swiss population (OR 234.9 95% CI 91.18–605.2, *p* < 0.001). All five patients had GPA, whereas no cases with EGPA or MPA were identified. Anti-PR3 antibodies were detected in four out of five patients, anti-MPO in one patient. Since LVV can occur in a significant proportion of patients with GPA, evaluation for LVV may be considered systematically in the diagnostic workup of AAV.

## Introduction

According to the 2012 revised international Chapel Hill consensus conference nomenclature of vasculitides, antineutrophil cytoplasmic antibody (ANCA)-associated vasculitis (AAV) are small vessels vasculitis, encompassing granulomatosis with polyangiitis (GPA, previously known as Wegener's granulomatosis), microscopic polyangiitis (MPA), eosinophilic granulomatosis with polyangiitis (EGPA, previously known as Churg–Strauss disease), and single-organ AAV (like renal-limited AAV) [1]. Diseases with large vessel vasculitis (LVV) include Takayasu arteritis and giant cell arteritis (GCA, Horton’s disease), the first condition being much rarer than the latter.

The association between AAV and LVV is not clearly establish in the literature. Some authors refer to *polyangiitis overlap* syndromes, a type of systemic vasculitis that represents several diagnostic categories [2, 3]. Indeed, there are several reports of patients diagnosed with both GCA and GPA [2–13], MPA [7, 14–18], or EGPA [19]. A retrospective cohort of the Mayo Clinic identified five cases of temporal arteritis among 345 patients with GPA [4]. Another study found seven patients with necrotizing vasculitis among 120 temporal artery biopsies with GCA, including one case of GPA and three cases of MPA [7]. On the other hand, case series reports the presence of ANCAs in up to 30% of GCA patients, mostly without -PR3/-MPO specificity and without fulfilling the criteria for AAV [20, 21]. In the present study, we assessed the prevalence of LVV in AAV patients, by studying a cohort of 101 patients with established GPA, MPA, and EGPA.

## Patient data collection, materials and methods

We retrospectively selected patients from our hospital between 2006 and 2016 with the diagnosis of GPA, MPA, EGPA, and AAV based on medical records.

Patients were selected between 2006 and 2016 by a diagnostic code entered in the consultation or hospital discharge reports, as well as by an internal register of our department, and a list of positive ANCA results with MPO or PR3 specificity. Only patients who fulfilled the ACR criteria for GPA and EGPA, and the Chapel Hill definition for MPA were enrolled [1, 22, 23]. For GPA, at least two of the following four criteria must be present: (1) nasal or oral inflammation, (2) abnormal chest radiographic findings, (3) abnormal urinary sediment, (4) granulomatous inflammation in the biopsy specimen. For EGPA, at least four of the following six criteria must be present: (1) asthma, (2) eosinophilia > 10%, (3) mono or poly neuropathy (4) non fixed pulmonary infiltrate, (5) paranasal sinus abnormality, (6) extravascular eosinophils. For MPA, Chapel Hill definition was used: necrotizing vasculitis, with few or no immune deposits, predominantly affecting small vessels (i.e., capillaries, venules, or arterioles) with necrotizing glomerulonephritis or pulmonary capillaritis without granuloma formation. Patients without the above mentioned criteria or insufficient data were excluded from the study. We identified a fourth group, single-organ AAV with histologic evidence of small vessel vasculitis and specific ANCA (-PR3 or -MPO), but without sufficient elements for GPA, EGPA or MPA. However, since this group of AAV is still controversial, we excluded it from the current analysis.

Temporal arteritis or aortitis were defined by the presence of inflammatory infiltrates upon histology or by comparable imaging evidence (e.g., hypercaptation on PET-CT scans). Alternatively, a typical clinic of temporal arteritis according to the ACR criteria of 1990 was also retained [24].

This study was approved by the local ethics committee (GE 2016–00,040) with a waiver for the requirement of informed consent considering the retrospective nature of the study.

Clinical and biological data were collected from centralized electronic patient files and included demographic characteristics (age, gender, year of diagnosis), laboratory results (presence and subtype of ANCA), clinical characteristics (ENT, lung, renal, cardiac, eye, joint or skin involvement), cardiovascular risk factors (hypertension, dyslipidemia, tobacco, diabetes), cardiovascular morbidity (history of myocardial infarcts, stroke, heart disease, left ventricular ejection fraction), oncologic morbidity (history of cancer), treatments (acute and maintenance), and relapse rate. ANCA were analyzed by indirect immunofluorescence on NOVA Lite ANCA ethanol and formalin slides obtained from Inova Diagnostics, San Diego, USA, according to the manufacturer's instructions. Serum dilution started at 1/20. In case of positivity or doubtful results, an ELISA test on a DYNEX DSX processing system was used (Quanta Lite MPO/PR3 IgG ELISA, Inova Diagnostics, San Diego, USA) for confirmation, considering values < 20 units as negative. We also assessed serum creatinine, CRP, sedimentation rate at time of diagnosis. Finally, we analyzed the histological feature of biopsies if available. Most patients (89 of 108) had a follow-up of 3 years average duration, whereas 19 patients had only one visit and were lost to follow-up.

Results are reported as mean (± standard deviation) or median (± interquartile range (IQR)) for continuous variables, and in percentages (absolute numbers) for the categorical variables. The Clopper–Pearson statistical method was used to calculate the prevalence, while Mann–Whitney testing was employed to compare age between the AAV/LVV and non-LVV groups; Fisher’s exact test was used for all other analysis. Results were considered statistically significant if the *p* value was < 0.05.

## Results

**Fig. 1:**
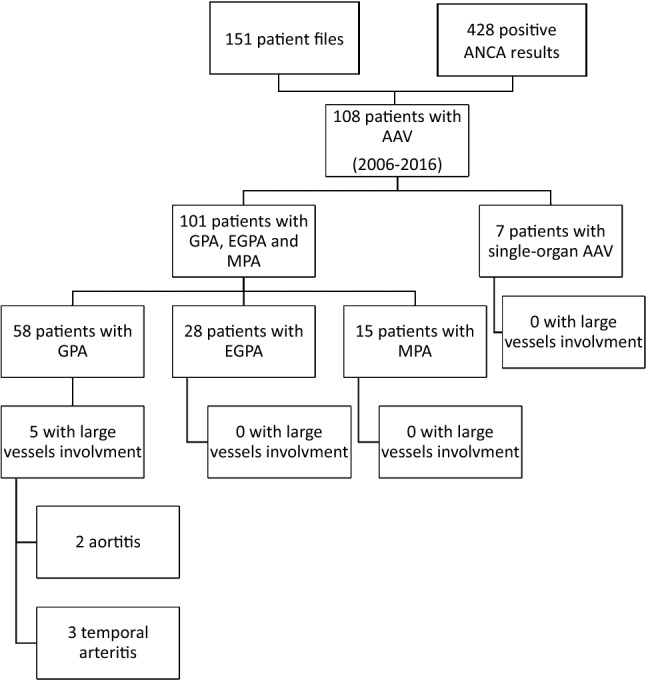
Retrospectively selected patients in the study. *AAV* ANCA-associated vasculitis, *GPA* granulomatosis with polyangiitis, *EGPA* eosinophilic granulomatosis with polyangiitis, *MPA* microscopic polyangiitis, *LVV* large vessel vasculitis

A total of 151 patient files and 428 positive ANCA results were identified. We selected 101 patients who fulfilled the ACR criteria or the Chapel Hill definition for GPA, EGPA, and MPA vasculitis. Seven supplementary cases had single-organ AAV, but were not further analyzed (Fig. 1).

### Demographic and clinical characteristics of patients

The median age at the diagnosis was 59 (33–85) (Table 1) with 41% of men and 59% of women. The ANCA specificities were uniformly distributed, with 38% of perinuclear ANCA (pANCA), 32% of cytoplasmic ANCA (cANCA), 34% of MPO, and 36% of PR3. As expected, cANCA-PR3 were more common in the GPA group (62%) and pANCA-MPO were more common in the MPA group (80%). EGPA had just over 40% of ANCA positive. The most frequent clinical manifestation was pulmonary (71%), followed by ENT (58%), then renal (53%), and joint (36%) involvement.

**Table 1:** Demographic and clinical characteristics of the patients with ANCA-associated vasculitis

Characteristics	GPA group (*n = *58)	EGPA group (*n = *28)	MPA group (*n = *15)	Single-organ AAV group (*n = *7)	Overall (*n = *108)
Demography					
Age at diagnosis – yr					
Median (IQR)	60(24.8)	50(25.5)	69(20.5)	70(8.0)	59(26.0)
Sex—*n* (%)					
Male	25(43)	11(39)	5(33)	3(43)	44(41)
Female	33(57)	17(61)	10(67)	4(57)	64(59)
Laboratory—*n* (%)					
pANCA	13(22)	10(36)	13(87)	5(71)	41(38)
cANCA	30(52)	2(7)	1(7)	2(29)	35(32)
xANCA	4(7)	1(4)	0(0)	0(0)	5(5)
MPO	9(16)	11(39)	12(80)	5(71)	37(34)
PR3	36(62)	0(0)	1(7)	2(29)	39(36)
Clinical manifestations—*n* (%)					
ENT	44(76)	19(68)	1(7)	0(0)	63(58)
Ear	17(29)	2(7)	0(0)	0(0)	19(18)
Nose and sinus	41(71)	19(68)	1(7)	0(0)	61(56)
Throat	10(17)	0(0)	0(0)	0(0)	10(9)
Renal	29(50)	6(21)	15(100)	7(100)	57(53)
Pulmonary	36(62)	27(96)	10(67)	4(57)	77(71)
Nodule	20(34)	3(11)	4(27)	0(0)	24(22)
Fixed infiltrate	13(22)	13(46)	7(47)	3(43)	25(23)
Cavities	4(7)	0(0)	0(0)	0(0)	4(4)
Asthma	1(2)	21(75)	2(13)	0(0)	24(22)
Cardiac^a^	0(0)	9(32)	0(0)	0(0)	9(8)
Neurologic	20(34)	11(39)	4(27)	0(0)	35(32)
Peripheral	19(33)	6(21)	4(27)	0(0)	29(27)
Central	3(5)	5(18)	1(7)	0(0)	9(8)
Joint	27(47)	6(21)	5(33)	1(14)	39(36)
Skin	6(10)	7(25)	2(13)	0(0)	15(14)
LV involment—*n* (%)	**5(9)**	**0(0)**	**0(0)**	**0(0)**	5(5)*
Aortitis	2(3)	0(0)	0(0)	0(0)	2(2)
Temporal arteritis	3(5)	0(0)	0(0)	0(0)	3(3)

*GPA* granulomatosis with polyangiitis, *EGPA* eosinophilic granulomatosis with polyangiitis, *MPA* microscopic polyangiitis, *ENT* ear, nose, and throat, *LV* large vessel, *IQR* interquartile range

^a^Only related to vasculitis

^*^Involvement of large vessels found in 5 of the 101 patients with GPA, EGPA, and MPA (single-organ AAV non-included), corresponding to the prevalence of 5.0% [1.6–11.2%]

### Prevalence of large vessel involvement

Aortitis was found in two patients and temporal arteritis in three patients with GPA. No aortitis or temporal arteritis were identified in patients with EGPA or MPA. Therefore, LVV was found in 5 of the 101 patients analyzed, corresponding to the hospital-based prevalence of 5.0% [95% CI 1.6–11.2%] of our overall cohort, or in 5/58 (9%) patients with GPA. These values are clearly higher compared to the prevalence of GCA in a general population close to Switzerland (Freiburg im Breisgau in Germany with 222 cases per million inhabitants [25]), resulting in odds ratio of 234.9 (95% CI 91.18–605.2, *p < *0.001).

### Report of cases

Case 1: A 66-year-old woman presented with chronic bloody nasal discharge, sero-mucous otitis and acute arthritis of the right elbow. With the onset of episcleritis and the finding of cANCA-PR3, a diagnosis of GPA was made. The patient was treated with prednisone and cyclophosphamide, subsequently replaced by mycophenolate mofetil because of hemorrhagic cystitis. The evolution was favorable. Due to weight loss of 10 kg a thoraco-abdominal CT was performed 10 years later revealing hyperdense spontaneous thickening with enhancement around the ascending and horizontal aorta. In addition pulmonary opacity of the right lower lobe was detected and a diagnosis of mononeuropathy with falling foot was made. A PET-CT showed hypermetabolism (SUV max 8.1) at the aortic arch and the ascending aorta. cANCA-PR3 were present at all times. In this context, immunosuppression by corticosteroids and mycophenolate mofetil was intensified. PET-CT scanning 18 months later showed a clear decrease in hypercaptation.

Case 2: A 52-year-old woman known for intracranial hypertension for 7 years, who was hospitalized for the investigation of headaches with tonic epileptic seizures. A brain MRI showed diffuse pachymeningitis. Due to unusual headaches located in the temporal area associated with hyperesthesia of the scalp and inflammatory syndrome, a temporal artery biopsy was performed and showed inflammatory lymphocytic infiltrate with intimal fibrosis, disappearance of the elastic lamina, and vasa vasorum granulomas. Nasal biopsy revealed mixed inflammation with rough granuloma. With MRI pansinusitis and positive cANCA-PR3, the diagnosis of GPA was made, and the patient was treated with corticosteroids and rituximab with an improvement in inflammatory syndrome and symptoms.

**Fig. 2:**
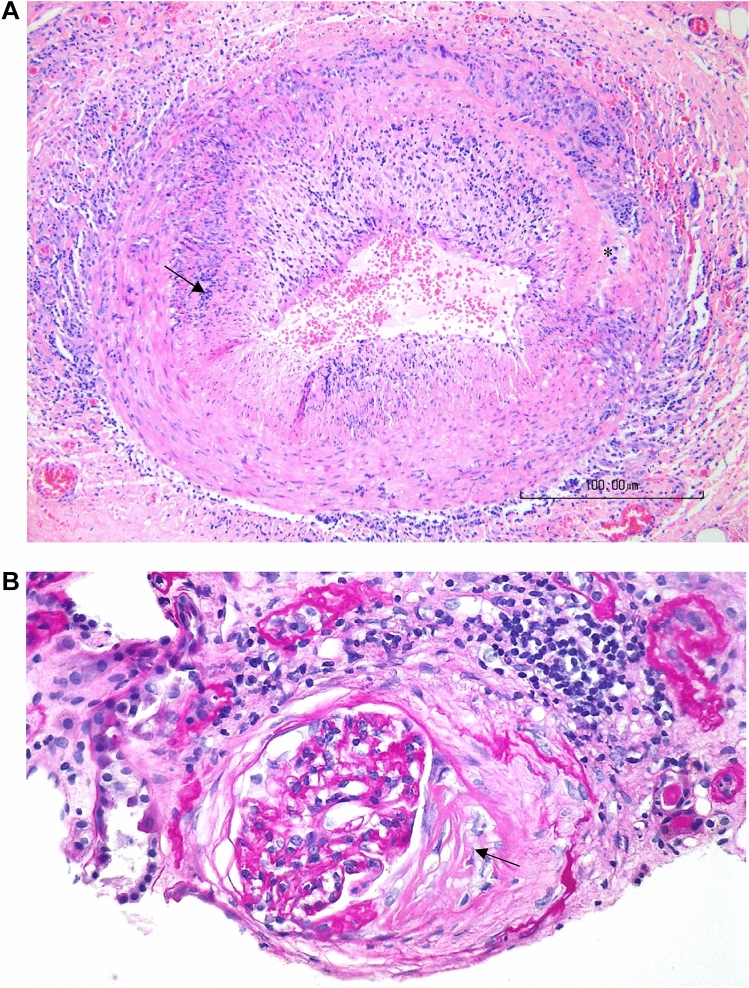
An 82-year-old patient diagnosed with granulomatosis with polyangiitis (sinusitis, destruction of nasal cartilage, subglottic stenosis, vertigo, glomerular hematuria, pANCA-MPO), and giant cell temporal arteritis. **A**: temporal arteritis with granulomatous inflammation with giant cells (arrow) and rupture of the internal elastic lamina (*) (courtesy of Jean-Claude Pache, MD). **B**: chronic glomerulonephritis with an extra-capillary fibrous crescent (arrow) (courtesy of Solange Moll, MD)

Case 3: A 76-year-old woman presented with progressive bilateral headaches and jaw claudication. The laboratory results revealed inflammation with a CRP of 120 mg/l and a sedimentation rate of 67 mm/h. A temporal biopsy showed a granulomatous and giant cell vasculitis (Fig. 2A). However, due to subglottic stenosis, nasal cartilage destruction, MRI pansinusitis, and persistent glomerular hematuria, a renal biopsy was performed showing segmental fibrous crescents compatible with pauciimmune extra-capillary glomerulonephritis (Fig. 2B). Perinuclear ANCAs were found, specifically anti-MPO. The perinuclear ANCAs remained positive over a 5-year follow-up whereas anti-MPO titers disappeared under treatment.

Case 4: A 63-year-old patient known for GPA with crusty rhinitis, CT-scan lung nodules, orbital mass and positive cANCA-PR3 was diagnosed with GCA based on ACR criteria 1990 with intermittent jaw claudication, hypersensitivity of the scalp, and transient loss of vision. A temporal biopsy was negative. However, his symptoms responded well to treatment with corticosteroids (prednisone 1 mg/kg) with normalization of erythrocyte sedimentation rate and methotrexate for a total duration of 4 years. Following the appearance of an orbital pseudotumor with diplopia, treatment with rituximab was able to induce and maintain remission.

**Fig. 3:**
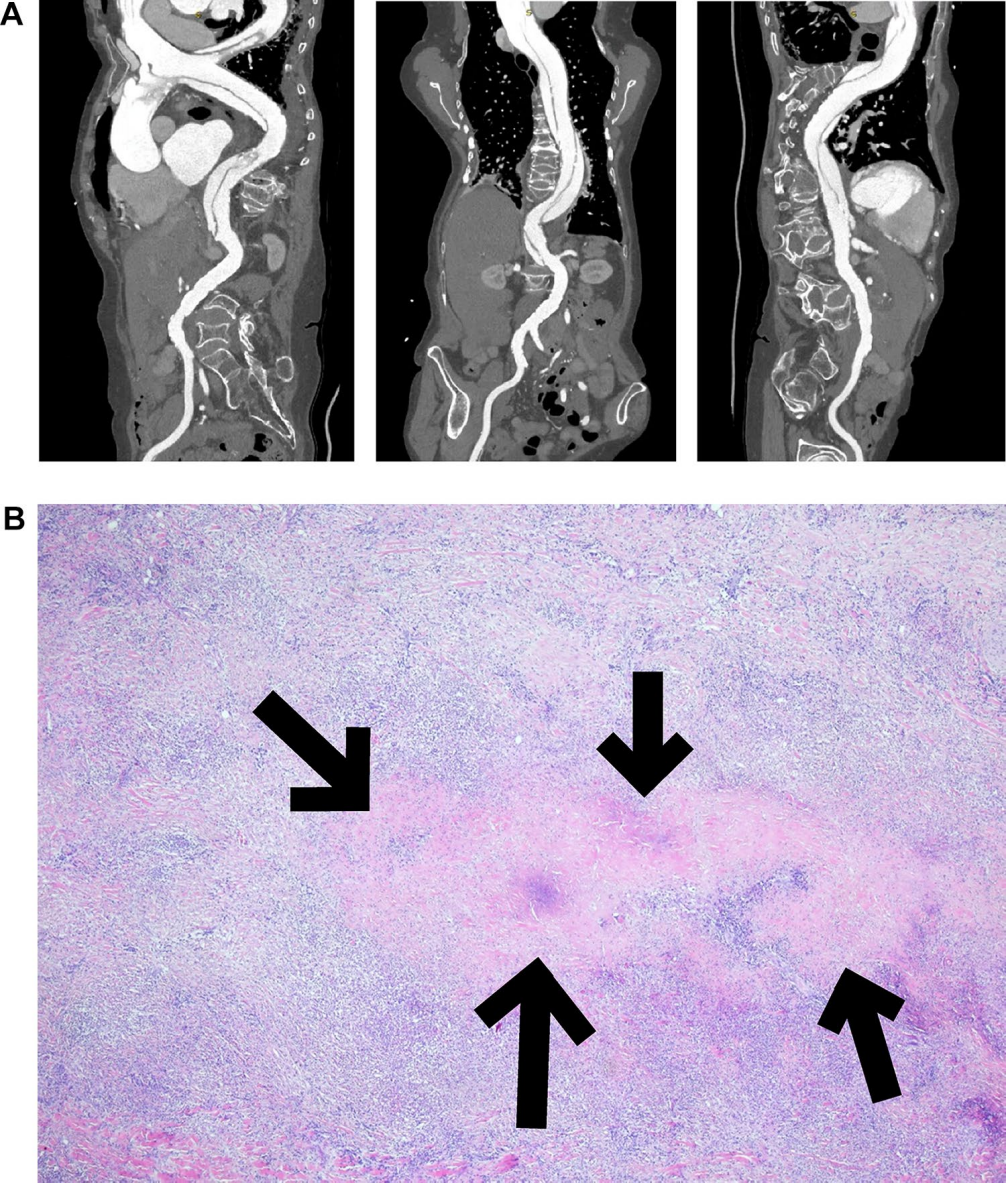
A 46-year-old woman with GPA diagnosis who presents a type A aortic dissection 24 years later. **A**: CT-scan showing aortic dissection, **B**: granulomatous vasculitis with chronic inflammation, multiple focus of collagenous necrosis (arrows) without giant cells (courtesy of Jean-Claude Pache, MD)

Case 5: A 46-year-old woman, known for GPA with nasal polyps, lower limb neuropathy, and optic neuritis with granulomatous ocular infiltration, presented with aortic dissection of type A (Fig. 3A). She benefited from an emergency operation with replacement of the ascending aorta. The pathological analysis showed a granulomatous vasculitis with chronic inflammation and multiple foci of collagenous necrosis without giant cells (Fig. 3B). The evolution was satisfactory after the operation under treatment with corticosteroids.

The clinical characteristics of the five AAV patients with LVV are summarized in Table 2.

**Table 2:** Demographic and clinical characteristics of the ANCA-associated vasculitis patients with large vessel vasculitis involvement (next page)

Characteristics	Patient I	Patient 2	Patient 3	Patient 4	Patient 5
Demography					
Age at diagnosis (years)	66	52	76	63	46
Sex	F	F	F	M	F
Diagnosis					
ANCA associated vasculitis	GPA	GPA	GPA	GPA	GPA
Large-vessels vasculitis	Aortitis	Temporal arteritis	Temporal arteritis	Temporal arteritis	Aortitis
Clinical manifestations					
ENT	Sinusitis, SMO	Sinusitis	NCD, SGS	Crusts, OPT	Nasal polyps
Renal	–	Hematuria	Hematuria	–	–
Pulmonary	Nodules/infiltrate	–	Nodules	Nodule	–
Neurologic	–	Pachymeningitis	–	–	Optic neuritis
Joints	Arthritis	–	–	–	–
Cardiovascular risk factors	Hypertension, tobacco	Dyslipidemia, tobacco, DM	Hypertension, tobacco	Hypertension, tobacco	Dyslipidemia
Laboratory					
ANCA	cANCA-PR3	cANCA-PR3	pANCA-MPO	cANCA-PR3	cANCA-PR3
Creatinin at diagnosis (mmol/l)	–	42	59	–	–
CRP at diagnosis (mg/l)	–	176	120	–	–
Sedimentation rate at diagnosis (mm/h)	–	85	67	–	–
Histology	Nasal( − )	Nasal( – ), TA( +)	Renal( +), TA( +)	TA( – )*	Eye( +), Aorta( +)
Treatments					
Induction	Cs + Cp	Cs + Rtx	Cs + Mtx	Cs + Mtx	Cs + Cp
Relapse	Cs + Rtx	–	–	Cs + Rtx	Cs + Rtx

*M* male, *F* female, *GPA* granulomatosis with polyangiitis, *SMO* sero-mucous otitis, *NCD* nasal cartilage destruction, *SGS* subglottic stenosis, *OPT* orbit pseudotumor, *DM* diabetes mellitus, *DL* dyslipidemia, *TA* temporal artery, *Rtx* rituximab, *Cs* corticosteroids, *Mtx* methotrexate, *Cp *cyclophosphamide

^*^Typical symptoms with jaw claudication, hyperesthesia of the scalp and pains of the belts

### Comparison of AAV patients with and without large vessel involvement

**Fig. 4:**
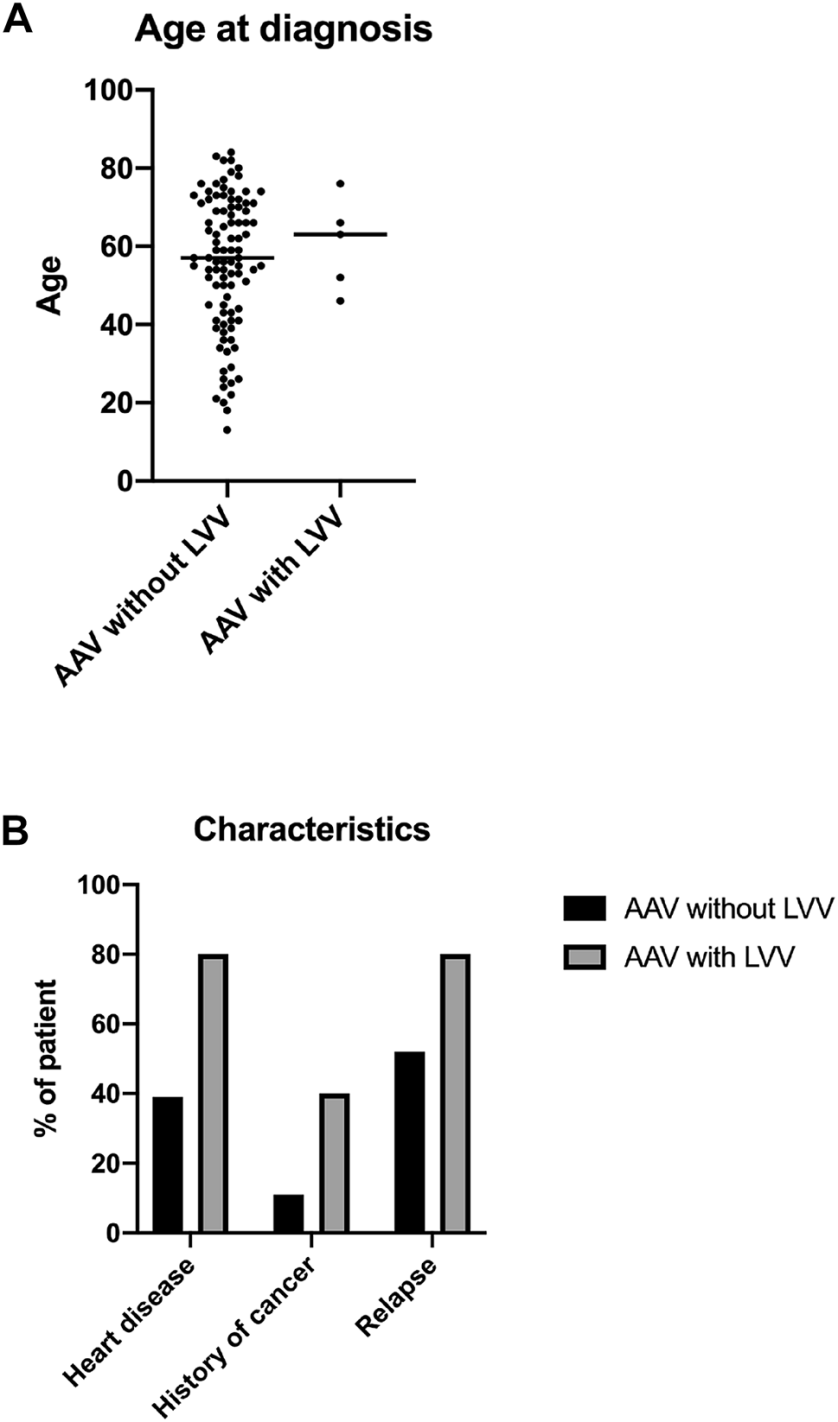
**A** Age of ANCA-associated vasculitis (AAV) patients with and without large vessel vasculitis (LVV) (63 ± 14.0 versus 57 ± 27.5 years *p = *0.65). **B** Prevalence of heart disease (80 versus 39%), history of cancer (40 versus 11%), and relapse (80 versus 52%) of AAV patients with and without LVV

Table 3 summarizes the differences between AAV patients with and without large vessel involvement. It is of note, that neither of these observations reached statistical significance. The age was not different with 63 ± 14.0 versus 57 ± 27.5 years (*p = *0.65) (Fig. 4A), and the sex ratio was similar, with slightly more women than men in both groups. At the laboratory level, more anti-PR3 than anti-MPO (60 versus 34%) were found, a finding at the limit of statistical significance (*p = *0.06). Among the five AAV patients with LVV, all having GPA, ENT manifestations seemed to be more predominant (100%), with less renal, pulmonary, neurological, and joint involvement. In the AAV group without LVV, lung manifestations (73%) were more frequent than of ENT manifestations (60%), and renal (50%) impairment (Table 3). Cardiovascular risk factors appeared to be more prevalent in the LVV group than the group without LVV, for arterial hypertension (OR 2.29 [0.45–13.26] *p = *0.39), dyslipidemia (OR 1.70 [0.29–8.67] *p = *0.62), and smoking (OR 6.39 [0.97–79.13] *p = *0.16). Finally, there was more cardiac disease of any cause (hypertensive, rhythmic, valvular, ischemic, dilated, restrictive) (OR 6.37 [0.97–79.13] *p = *0.15), and more cancer (OR 3.09 [0.55–18.20] *p = *0.21) in the group with large vessel involvement (Fig. 4B). There was no major difference in treatment, consisting mainly of corticosteroids (100 versus 93%), cyclophosphamide (40 versus 54%), and rituximab (20 versus 15%). Relapse was slightly more common in the LVV group (OR 3.68 [0.57–45.88] *p = *0.37).

**Table 3:** Demographic and clinical characteristics of the patients with or without large vessel vasculitis (LVV) in granulomatous polyangiitis (GPA), eosinophilic granulomatosis with polyangiitis (EGPA), and microscopic polyangiitis (MPA) patients*

Characteristics	AAV with LVV group (*n = *5)	AAV without LVV group (*n = *96)	*P* value	Odds ratio (95% CI)
Demography				
Age at diagnosis**—**yr				
Median (IQR)	63(14.0)	57(27.5)	0.65	
Sex**—***n* (%)				
Male	1(20)	40(42)	0.65	0.35(0.03–2.27)
Female	4(80)	56(58)	0.65	2.86(0.44–35.7)
Laboratory—*n* (%)				
pANCA	1(20)	35(36)	0.65	0.44(0.03–2.83)
cANCA	3(60)	29(30)	0.32	3.46(0.67–20.05)
xANCA	0(0)	5(5)	1.00	0.00(0.00–18.89)
MPO	1(20)	31(32)	1.00	0.52(0.04–3.42)
PR3	3(60)	33(34)	0.06	7.63(1.16–94.62)
Clinical manifestations—(%)				
ENT	5(100)	58(60)	0.15	∞(0.90-∞)
Renal	3(60)	48(50)	1.00	1.50(0.29–8.71)
Pulmonary	3(60)	70(73)	0.62	0.56(1.11–9.14)
Cardiac	0(0)	9(9)		
Neurologic	2(40)	33(34)	0.34	2.86(0.56–16.57)
Joint	1(20)	37(39)	0.38	2.39(0.47–13.85)
Skin	0(0)	15(16)	0.59	0.00(0.00–3.54)
Cardiovascular risk factors—*n* (%)				
Hypertension	3(60)	38(40)	0.39	2.29(0.45–13.26)
Dyslipidemia	2(40)	27(28)	0.62	1.70(0.29–8.67)
Tobacco	4(80)	37(39)	0.16	6.39(0.97–79.13)
Diabetes	1(20)	14(15)	0.56	1.46(0.11–10.15)
Cardiovascular morbidity—*n* (%)				
History of myocardial infarcts	1(20)	12(13)	0.51	1.75(0.13–12.39)
Priors of stroke	0(0)	12(13)	1.00	0.00(0.00–5.54)
Cardiac disease	4(80)	37(39)	0.15	6.37(0.97–79.13)
LVEF < 65%	1(20)	16(17)	1.00	1.25(0.10–8.53)
Oncologic morbidity—*n* (%)				
History of cancer	2(40)	11(11)	0.21	3.09(0.55–18.20)
Treatments—*n* (%)				
Glucocorticoids	5(100)	89(93)	1.00	∞(0.09-∞)
Cyclophosphamide	2(40)	52(54)	0.66	0.56(0.10–2.88)
Rituximab	1(20)	14(15)	0.56	1.46(0.11–10.15)
Relapse—*n* (%)	4(80)	50(52)	0.37	3.68(0.57–45.88)

^*^Single-organ AAV patients excluded

PET-CT was performed in 12 of the 101 patients and positive for LVV in one case (patient 1). However, most patients (74/101) had an injected thoracic CT-scan which makes it possible to detect vasculitis of the large vessels by wall thickening and uptake of contrast medium of the arterial wall. Of the remaining patients, 12 of the 27 patients had a cardiac ultrasound. Three temporal artery biopsies were performed when typical clinical symptoms of temporal arteritis were present (2/3 positive). In one case, histological examination was performed following aortic replacement (patient 5).

## Discussion

The overlap between LVV and AAV is rare. Nevertheless, we found a prevalence of 5.0% [95% CI 1.6–11.2%] in our cohort of 101 patients. Beside several case reports, there is little systematic data available. Nishino et al. reported five cases of temporal arteritis in a cohort of 345 patients with GPA, corresponding to a prevalence of only 1.4% [4]. Conversely, a more recent French retrospective study analyzed 120 patients with a diagnosis of GCA and identified 7 patients (5.8%) with necrotizing vasculitis, polyarteritis nodosa in two cases, one GPA, three MPA and an unclassified systemic vasculitis [7]. Furthermore, Delaval et al. recently described 50 cases of temporal arteritis with features of AAV as compared to controls with classic GCA in a retrospective case–control study [26]. Atypical symptoms for GCA with ENT involvement was present in 32% of the patients, followed by renal (26%), pulmonary (20%) and neurologic (16%) involvement. Moreover, fibrinoid necrosis and small branch vasculitis was found in 23% of these patients but in none of the controls with classic GCA. The authors, therefore, proposed to actively search for AAV in patients suffering from temporal arteritis with atypical clinical symptoms, a suggestive histology (fibrinoid necrosis), or resistance to treatment. A review of the literature published in 2018 searching for case reports of GPA with concomitant cephalic GCA (e.g., temporal arteritis), reported a total of 15 cases [8]. Unfortunately, the data were heterogeneous, unlike our study, not all patients had ANCA blood testing. In addition, not all patients had biopsies compatible with both entities. Interestingly, in seven cases, GCA preceded GPA while in three cases GPA was diagnosed before GCA. Four patients had GPA and GCA concomitantly. In our study, two patients had GPA before GCA (with aortitis), and three had GPA and GCA concurrently (with temporal arteritis).

Skeik N et al. report ten cases of aortitis related to AAV, in addition to their case report, and suggested using rituximab for treatment [27]. By studying 16 consecutive patients with periaortitis diagnosed by CT scanning, Vaglio et al. identified three cases with positive ANCAs including one with crescentic necrotizing glomerulonephritis [28]. Finally, 16 GPA patients with aortitis, aortic dissection and/or aneurysm were reported in a recent study from China [29].

The prevalence of LVV in our study was significantly higher than that of the southern German population. However, it is important to emphasize that this hospital-based prevalence is not directly comparable to a population-based prevalence.

The pathophysiologic mechanisms of large vessels involvement in AAV are largely unknown. While some authors speculate on invasive inflammatory cells and granulomas causing aortitis, others believe that inflammation results from necrotizing vasculitis involving the vasa vasorum of the aortic wall (like in Takayasu disease) [30]. But the most intriguing question is whether large vessel involvement in AAV is a coincidental overlap of AAV–GCA/AAV–Takayasu disease or whether LVV belongs to the spectrum of AAV. Chirinos JA et al. support the second hypothesis based on a review of the literature with clear epidemiologic, clinical and pathologic differences between large-vessel AAV on one side and GCA/Takayasu on the other side [31]. However, this point remains a matter of debate.

Analysis of the demographic data of AAV patients with large-vessel involvement does not show any clear differences. ENT manifestations seemed to be more common, not surprisingly since all cases with LVV were identified among patients with GPA. Investigation of cardiovascular risk factors revealed a trend towards a higher prevalence of hypertension, dyslipidemia, and tobacco in the LVV group. Moreover, we found more cardiac disease of any type (hypertensive, valvular, rhythmic, ischemic, dilated or constrictive) in the LVV group (OR 6.37 [0.97–79.13] *p = *0.15). This could be explained either by a link between GCA and cardiovascular risk factors, there was no age difference. The trend for a higher slightly higher prevalence of cancers (3.09 [0.55–18.20] *p = *0.21) can also not be explained by age. Finally, we note that LVV patients have slightly more relapse. Unfortunately, our study did not have the statistical power to detect statistically significant differences between the groups with and without involvement of the large vessels. Thus, it is difficult to draw firm conclusions and studies with more cases are needed to corroborate our results.

Our study has some limitations. First to mention is the small overall number of AAV patients with LVV which causes a significant risk of analysis bias. Second, the group of LVV patients is not homogeneous. We have no explanation why we did not identify any LVV patients associated to MPA or EGPA, even though several such cases are described in the literature [2, 7, 14–19]. Third, not all patients had the same diagnostic approach using different methods of investigation. Patient 1 was diagnosed with LVV based on PET-CT imaging results, while in patients 2, 3 and 5, LVV diagnosis was based on biopsy samples, and in patient 4 a diagnosis of GCA was made based on ACR criteria. However, the diagnostic approach for LVV is currently changing with an increasing role of imaging studies and a diminished role of biopsy studies. Hence, increasing use of imaging studies may explain the increase in LVV prevalence. Finally, only approximately 70% of the patients underwent large vessels imaging. Consequently, LVV might not have been detected in some of the remaining patients potentially underestimating, therefore, the overall prevalence.

It remains controversial to systematically recommend large vessel evaluation in all AAV patients. Nevertheless, proactive history taking and careful physical examination for signs or symptoms suggestive of LVV are required. Furthermore, considering the presence of LVV in approximately 1 out of 20 patients with AAV and the potential morbidity associated with aneurysms for instance, it seems reasonable to evaluate existing imaging studies (US, MRI, CT scans, echocardiography) or to request additional investigations in the search for LVV. However, this question needs to be addressed in a large multi-center study.

## Conclusion

Large-vessels involvement was found in five AAV patients (two with aortitis and three with temporal arteritis) in our cohort of 101 patients with GPA, EGPA, MPA, resulting in a hospital-based prevalence of 5.0%. According to the clinical importance of this co-existing diseases, evaluation for LVV may be considered systematically in the future diagnostic workup of AAV.
